# Distribution of β-Glucan, Phenolic Acids, and Proteins as Functional Phytonutrients of Hull-Less Barley Grain

**DOI:** 10.3390/foods8120680

**Published:** 2019-12-13

**Authors:** Gordana Šimić, Daniela Horvat, Alojzije Lalić, Daliborka Koceva Komlenić, Ivan Abičić, Zvonimir Zdunić

**Affiliations:** 1 Agricultural Institute Osijek, 31000 Osijek, Croatia; gordana.simic@poljinos.hr (G.Š.); alojzije.lalic@poljinos.hr (A.L.); ivan.abicic@poljinos.hr (I.A.); zvonimir.zdunic@poljinos.hr (Z.Z.); 2 Faculty of Food Technology Osijek, Josip Juraj Strossmayer University of Osijek, 31000 Osijek, Croatia; daliborka.koceva@ptfos.hr

**Keywords:** hull-less barley, β-glucan content, hordeins, phenolic acids, antioxidant activity, roller milling

## Abstract

Two hull-less barley varieties were roller-milled, and breaks (B) and reduction flours (C), shorts, and bran were collected. Shorts, which mainly originate from endosperm cells with a smaller amount of the outer layers, had the largest yield (48.87–51.54%). Ash (0.82–3.10%) and protein (9.95–14.8%) increased from flours toward shorts and bran, while starch decreased (82.31–48.69%). In contrast to clear distribution differences in protein content (bran > shorts > C > B), albumins/globulins content was lowest in bran (0.78–0.90 g/100 g_dw_), and their distribution between fractions was uneven and genotype dependent. Distribution of hordeins (6.69–10.49 g/100 g_dw_) was more distinct and generally decreased in order from bran > B > shorts > C. The proportion of nutritionally poor C-hordeins in total hordeins varied from 28.33% to 30.24%, without significant differences between fractions. The β-glucan content varied from 0.80% to 7.49% with decreasing content in the order bran, shorts > C > B. Shorts and bran could be classified as moderate and high β-glucan flour (5.70–7.22%). The total phenolic and antioxidant activities ranged from 0.91 to 2.21 mg GAE/g_dw_ and 28.81–72.06%, respectively. Ferulic and sinapic acids determined by high-performance liquid chromatography (HPLC) were major contributors to the antioxidant activity (45.16–1026.91 ug/g_dw_ and 18.93–206.52 ug/g_dw_), respectively. The yield and high content of phytonutrients make hull-less barley shorts suitable for the production of health-promoting food and food supplements.

## 1. Introduction

Barley is an important cereal crop, globally grown for animal feed use and as a source of fermentable sugars in brewing and distilled beverages production [1]. Barley was one of the first cereals recognised as a food that provides vital nutrients and energy to maintain body functions and health. Cultivation of barley for food purposes represents only 2% of current global production, but in some countries with extreme climates, in Asia, Himalayan nations, and North Africa, it remains a staple cereal food source [2].

Barley is naturally high in dietary fibre, especially β-glucan and phenolic compounds, which have the potential to lower cholesterol and blood glucose levels and helps gut microbial balance [1,3,4]. Mixed linked β-D-glucans, together with arabinoxylans, are prevailing fibre constituents of the barley endosperm and aleurone cell walls [5]. It is a characteristic of barley that its β-glucan is distributed throughout the aleurone and endosperm rather than being confined to outer branny layers [6]. The β-glucan content of barley grain is mainly determined by genotype and less by environmental conditions during the grain filling period, but a significant influence of both genetic and location factors on barley β-glucan content was also noticed [2]. Significant differences in β-glucan content among barley types with various starch amylose contents were observed. The high-amylose starch type barleys contained higher amounts of β-glucan than the waxy, zero, and normal amylose endosperm types [7]. It was reported that hull-less atypical amylose varieties had a significantly higher concentration of β-glucan than the normal amylose ones. Further, the difference in β-glucan content between the hull-less and hulled varieties of normal starch type was not significant, but there was an observed tendency towards a higher β-glucan content in hull-less barley samples [5].

In addition to providing high amounts of dietary fibre, especially soluble β-glucan, barley grains are rich in protein, complex carbohydrates, minerals, and fat-soluble vitamin E (tocols) [1,8,9]. Due to the high content of proteins, the second major component of dry grain, barley is an affordable source of food proteins. According to their extractability characteristics (classification method developed by Osborne), barley grain proteins can be classified into four groups, i.e., albumins, globulins, hordeins, and glutelins. Albumins and globulins are mainly located in the bran and embryo, thus, called cytoplasmic proteins. They comprise 3–4% and 10–20% of the total seed proteins, respectively. Hordeins belong to prolamin protein fraction, an alcohol-extractable protein fraction of barley grain that accounts, on average, up to for up to 60% of the total grain nitrogen, which makes them the main barley endosperm storage proteins. In accordance with electrophoretic mobility and amino acid composition, the hordeins are classified into five groups of polypeptides, called A-hordein, B-hordein (70–90%), C-hordein (10–20%), D- and γ-hordein (less than 5%). Hordeins and glutelins are mainly found in the starchy endosperm, together contributing to about 70–90% of the total dry seed proteins [2,10,11,12].

Barley also contains significant content of phenolic compounds, naturally occurring antioxidants with antiradical and antiproliferative potentials [13,14,15]. Phenolic compounds constitute a large heterogeneous group of compounds that can be classified in different ways, based on their chemical structure, occurrence, and distribution in nature, and according to their location in the plant tissue [4,16]. Phenolic acids are the most abundant phenolic group of phytochemicals in barley, existing dominantly in the form bound to compounds of the cell wall, followed by conjugated and free forms. Free forms of phenolic acids in barley account for a small portion of the total phenolic acid concentration, and they are mainly ferulic acid, vanillic acid, syringic acid, and p-coumaric acid [13,17]. It was reported that phenolic acid concentration on dry basis in barley approximately ranges between 4.6 and 23 mg/g for the free form, between 86 and 198 mg/g for the conjugated form, and between 133 and 523 mg/g for the bound form, whereas the total phenolic acid concentration ranges between 604 and 1346 mg/g [18,19]. The most present phenolic acid found in barley grain is ferulic acid, accounting approximately 68% of total phenolic acids in barley, and its concentration in barley grains ranges between 149 and 413 mg/g [20,21]. Researchers have linked a diet high in polyphenols and their metabolites to the maintenance of the gut microbial balance and a reduced risk of developing some chronic diseases [4].

Cereal grains have a long history of usage for both human and animal consumption and as a raw material for industrial purposes. For consumption food, milling of grains and producing of the flours is often the first step in preparing daily meals in many parts of the world. Different milling processes applied are associated with nation consuming customs and characteristics of the cereals used to produce various mill streams. The milling process can basically be described as a sequence of procedures involving grinding, sifting, and separation and regrinding in order to separate particular parts of the grain [22]. The initial studies on roller milling of hull-less barley have often been designed to follow the already-installed procedure of wheat milling, focused on flour yield and flour composition [23,24]. More recently, several studies were dealing with hull-less barley milling in order to produce products other than standard flour which are enriched in valuable dietary fibre originating from endosperm cell walls and other bioactive phytochemicals. It has been brought to researchers’ attention that the coarse material coming out from the final reduction passage, appointed as “shorts” in wheat milling, originates mainly from endosperm cell walls in barley milling, and is enriched in β-glucans and other fibre constituents. To reflect its composition, and to distinguish it from bran fraction, this coarse fraction resulting from hull-less barley roller milling procedure was designated as a fibre-rich fraction [25,26,27]. Nowadays, consumers’ awareness of the need for healthier life habits is growing, and reformulation of a daily diet in terms of nutritional quality can certainly contribute. Hull-less barley has been increasingly researched because of its beneficial composition, which can be utilised in developing of functional food products. It is a cereal that could be easily milled to produce flours of different compositional characteristics using common milling systems. When compared to wheat, the milling processing of barley is still relatively uncommon and less developed to be widely recognised [27,28,29]. Supplementing wheat flour with hull-less barley, either as a whole grain or as a flour addition, especially when enriched in dietary fibre, phenolic compounds, and protein content, could help enhance the value of the basic raw material for traditional bakery recipes or diverse pasta products.

The present study was concerned with the milling of hull-less barley and the analytical characterisation of hull-less barley milling streams and flours. In addition to conventional flour and millstream characterisation mainly focusing on complete chemical analysis and health-promoting fibres (β-glucans), in this study, the distribution of important functional phytonutrients, such as phenolic acids and specific proteins, were determined.

## 2. Materials and Methods

### 2.1. Materials

Two hull-less barley varieties, Mandatar (recognised 2017) and Osvit (recognised 2014), created at the Agricultural Institute Osijek, were included in this study. They were sown as winter barley in an area of the Slavonia region (45.5550° N, 18.6955° E), which belongs to the eastern part of Croatia, characterised by a moderate continental climate. The field trials with hull-less barley were installed in fall of 2017, and grains were harvested in the early summer of 2018. During harvesting, the hull was easily separated from the grain. The naked grains were collected and stored until milling analysis. Physical characteristics of the grains were estimated. Samples were analysed for thousand kernel weight and hectolitre weight, accordingly.

### 2.2. Milling Procedure

The grain samples of hull-less barleys were subjected to the roller milling process using laboratory milling equipment. The flour research laboratory at the Faculty of Food Technology Osijek (Osijek, Croatia) uses Bühler MLU 202 laboratory mill (Bühler AG, Uzwill, Switzerland), which is mostly used for wheat samples testing. Samples for roller milling were prepared by weighing 3 kg of each hull-less barley. Prior to milling, the barley grains were cleaned to ensure the samples were free from any impurities and conditioned to 14.0%, *w*/*w* moisture content. Conditioning was performed in two steps. The first step was conditioning to 13.5%, *w*/*w* moisture content, by adding a calculated amount of water to the grain, thoroughly mixing to evenly distribute moisture throughout the grain, and storing it for 24 h in a sealed container at the room temperature. Leaving it overnight allows water to penetrate the kernel, and in order to adjust the intended moisture content of 14% (*w*/*w*), the remaining amount of water was added half an hour before milling. The mill uses a six-roll system to make three breaks (B1–B3), three reductions (C1–C3) of refined flours, bran, and shorts [30]. The flour fractions were separated from bran and shorts on sieves with following mesh openings: 710 (30), 600 (36W), 530 (40W), 180 (8XXX), and 150 µm (9XXX) and using preset feed rate and roll gap settings. Samples were prepared and milled as duplicates.

### 2.3. Composition of Hull-Less Barley Fractions

Ash content was determined by incineration in a muffle furnace at 550 °C for 3 h [31]. Protein content was determined according to the Kjeldahl method [32]; Kjeltec 2300 Analyser Unit, Foss Analytical AB, Höganäs, Sweden). Fat content was analysed by the Soxhlet extraction method. Starch content was determined according to the Ewers polarimetric method [33].

### 2.4. β-Glucan Content

The total β-glucan content was determined according to the method reported by Mc Cleary and Glennie [34] using a Megazyme mixed-linkage β-glucan assay kit (Megazyme Ltd., Bray, Ireland).

### 2.5. Extraction of Samples

Extracts for the determination of total phenolic content and antioxidant activity were prepared by weighing 2 g of hull-less barley flour fraction and mixed with 5 mL of acidified methanol (HCl/methanol, 1:100, *v*/*v*). The mixture was homogenised by vortex for 2 min, placed afterwards in a Sonorex Digitec ultrasonic bath (Model RK510H; Bandelin, Germany) at room temperature and sonicated for 1 h. It was then centrifuged at 9000 rpm for 5 min at 4 °C (Universal 320R; Hettich, Germany). After centrifugation, the supernatant was removed, and the extraction procedure of the residue was repeated twice, firstly with 5 mL of acidified methanol and then with 2 mL with a time of sonication of 30 min. The supernatants were pooled. To avoid oxidation, extracts (12 mL) were stored in the dark at −20 °C, and analyses were performed within the next 24 h. All samples were prepared and analysed as triplicates.

### 2.6. Total Phenolic Content (TPC)

The TPC in hull-less barley flour extracts was determined with Folin-Ciocalteu reagent using the method described by Singleton and Rossi [35], with some modifications. To 0.1 mL of the extract obtained above, 5 mL of ten-fold diluted Folin-Ciocalteu’s phenol reagent and 0.9 mL of distilled water were added. The mixture was shaken, mixed with 4 mL of 7.5% Na_2_CO_3_, and left for incubation for 2 h at room temperature in the dark. The absorbance was read at 765 nm. The absorbance value was measured with a UV/VIS-Spekol spectrophotometer (Analytik Jena AG, Jena, Germany). Total phenolic content was expressed as milligrams of gallic acid equivalents per gram of dry weight (mg GAE/g_dw_) using a calibration curve constructed with gallic acid (50–1000 µg/mL). All samples were analysed as triplicates.

### 2.7. Antioxidant Activity (DPPH Radical Scavenging Activity)

Antioxidant activity was measured using a modified version of the method explained by Brand-Williams, Cuvelier, and Berset [36]. This involved the use of free radical 2,2-diphenyl-1-picrylhydrazyl (DPPH) solution in the methanol. Every sample extract (0.2 mL) was reacted with 1 mL of a 0.5 mmol/L methanol solution of DPPH and 2 mL of methanol. The reaction mixture was shaken and incubated in the dark. The absorbance (A) of the solution was measured against a methanol blank at 517 nm after 30 min. Stable DPPH radical reaches the absorbance maximum at 517 nm, and its colour is purple. The change of this colour into yellow is a result of the pairing of an unpaired electron of a DPPH radical with the hydrogen of the antioxidant, thus generating reduced DPPH-H. Adding an antioxidant results in the decrease of absorbance, that is proportional to the concentration and antioxidant activity of the compound. Lower absorbance of the reaction mixture indicates higher free radical scavenging activity. Antioxidant activity was calculated as inhibition of free radical DPPH in per cent (**%)** by using the following equation:

% Antioxidant activity = (1 − (A of sample_t=30_/A of control_t=0_)) × 100

All samples were analysed as triplicates.

### 2.8. Extraction of Albumins/Globulins and Hordeins

Barley protein extractions were done according to the method of Celus et al. [37], with some modifications: for extraction of albumins and globulins, 50 mg of the sample with 1 mL of 4 M sodium chloride were vortexed for 5 min at 3500 rpm (VibroMix 204 EV, Tehtnica, Slovenia), incubated at 25 °C for 30 min at 500 rpm (Thermomixer 5436, Eppendorf, Germany) and centrifuged (Centrifuge 5415 C, Eppendorf, Germany) for 10 min at 14000 rpm. The supernatant was decanted, filtered in glass vials through 0.45 μm polyvinylidene fluoride (PVDF) membrane filter (Ahlstrom GmbH, Germany) and stored at −20 °C until analysis. Hordeins were extracted from the pellet residue by adding 1.0 mL of 50% 1-propanol + 1% dithiothreitol, vortexed for 5 min, incubated at 60 °C for 60 min, and centrifuged for 15 min. The supernatant was decanted, filtered in glass vials through 0.45 μm PVDF membrane filter and stored at −20 °C until analysis. All extractions were done as duplicates.

### 2.9. HPLC Analysis of Protein Fractions

Analysis of protein fractions was carried out using a Series 200 HPLC system (Perkin Elmer, Waltham, MA, USA) coupled with Discovery BioWide Pore RP-C18 column (4.6 × 150 mm, 300 Å, 5 μm). The mobile phase consisted of Millipore water with 1% trifluoroacetic acid (*v*/*v*) (A) and acetonitrile with 1% trifluoroacetic acid (*v*/*v*) (B). The column temperature was 50 °C and injection volume was 20 uL. The gradient elution profile was as follows: from 24% to 58% B in 30 min, isocratic at 90% B for 5 min, returning to the initial conditions in 5 min, and column equilibration in 5 min. The flow rate was controlled at 1.0 mL/min. The peaks were detected at 210 nm with a photodiode array detector. The groups of peaks under chromatograms belonging to D-, B-, C-hordeins and their proportion was expressed as a % of total hordeins area. For the calibration of HPLC absorbance area and the calculation of the albumins/globulins and total hordeins concentration, bovine serum albumin was used as a protein standard, and consequently, a calibration curve of five points (5–120 ug, *r* = 0.999) was constructed. All samples were analysed as duplicates.

### 2.10. Extraction of Phenolic Acids

The improved microscale method of Zavala‑Lopez and Garcia‑Lara [38] was used for phenolic acid extraction. Free phenolic acids were extracted as follows: 150 mg of hull-less barley flour fractions with 2.1 mL of 80% methanol were vortexed for 5 min at 3500 rpm (VibroMix 204 EV, Tehtnica, Slovenia). The sample was incubated at 25 °C for 15 min at 500 rpm (IKA KS 260 basic, Germany) and centrifuged (Universal 320R, Hettich, Germany) for 10 min at 5000 rpm. The supernatant was decanted and stored at −20 °C until analysis. The bound phenolic acids were extracted from the pellet residue as follows: 1.5 mL of 2 M NaOH was added and vortexed for 5 min at 3500 rpm. The alkaline hydrolysis was conducted at 90 °C for 1 h at 250 rpm (shaking water baths GFL 1092, Burgwedel, Germany). After hydrolysis, the sample was acidified with 1.5 mL of 2 M HCl at pH 2 (adjusted with 2 M HCl using pH strips). Lipids were removed by adding 2.4 mL of n-hexane to the samples, vortexed for 5 min at 3500 rpm, incubated at 25 °C for 10 min at 500 rpm, and centrifuged for 10 min at 5000 rpm. The hexane layer (upper phase) from the three-layered system was discarded. This procedure was repeated two more times. The bound phenolic acids were recovered by adding 2.4 mL of ethyl acetate, vortexed for 5 min at 3500 rpm, incubated at 25 °C for 10 min at 500 rpm, and centrifuged for 10 min at 5000 rpm. The ethyl acetate layer (upper phase) from the three-layered system formed was collected. The previous procedure with ethyl acetate washes was repeated two more times. The pooled ethyl acetate was evaporated to dryness (BÜCHI B-720 Vacuum Controller, Flawil, Switzerland), re-suspended in 600 μL of 50% methanol, and stored at −20 °C until analysis.

### 2.11. HPLC Analysis of Phenolic Acids

Analysis of free and bound phenolic acids was carried out using a Series 200 HPLC system (Perkin Elmer, Waltham, MA, USA) coupled with Kinetex Core-Shell RP-C18 column (150 × 4.6 mm, 100 Å, 5 um) according to Matilla et al., with some modifications [39]. Prior to analysis, samples were filtered through 0.2 μm nylon filter (Ahlstrom GmbH, Bärenstein, Germany). The mobile phase consisted of an A—Millipore water acidified with 1% trifluoroacetic acid (*v*/*v*) and B—acetonitrile acidified with 1% trifluoroacetic acid (*v*/*v*). The column temperature was 30 °C and injection volume was 20 uL. The gradient elution profile was as follows: from 5% to 40% B in 40 min, isocratic at 90% B for 5 min, returning to the initial conditions for 5 min and column equilibration for 5 min. The flow rate was controlled at 1.0 mL/min. The peaks were detected at 275 nm with a photodiode array detector. Peak identification was based on the retention times and spectral data of standards (190–400 nm). Calibration curves of five points were made by diluting stock standards (1 mg/mL) in methanol where all phenolic acids showed a linear response within the studied range (*R*^2^ > 0.999). All samples were prepared and analysed as duplicates.

### 2.12. Statistical Analysis

The experimental data were processed by one-way ANOVA using Statistica ver. 13.0 software (Stat Soft Inc., Tulsa, OK, USA) and PAST: paleontological statistics software package for PCA analysis. Significant differences among means (post-hoc tests) were calculated using Fisher’s least significant difference (LSD) test at the significance level of α = 0.05.

## 3. Results and Discussion

### 3.1. Hull-Less Barley Grain Characteristics

Two hull-less barley samples (Mandatar and Osvit) created at Agricultural Institute Osijek and grown in the eastern part of Croatia (Osijek city area) during vegetation period 2017–2018 were used. Soil type at the growing site is eutric cambisol with slightly acidic pH (pH = 6.5). Non-limiting levels of nutrients were applied prior to sowing with 50 kg ha^−1^ of UREA (46% of N), with subsequent application of 400 kg ha^−1^ of NPK fertiliser (formulation 7:20:30) as a starter and with additional fertilising just before awn emergence stage with 70 kg ha^−1^ of KAN (27% of N). Weeds and diseases were controlled as necessary. Weather data, including precipitation and temperature, were obtained from the AIO measuring station, which is located less than a kilometre from the growing site. Precipitation through the growing season (October 2017 till June 2018) was 426.8 mm with 23.3 °C mean maximum and −10.5 °C mean minimum temperature. Hull-less barley usually has hectolitre weight higher than standard hulled barley, and the results achieved for the varieties used in this study rated from 78 kg to 80 kg, for Osvit and Mandatar, respectively. The same grain samples were also analysed for thousand kernel weight, and the obtained results were as follows: 45.4 g for Mandatar and 48.0 g for Osvit variety.

### 3.2. Yield and Chemical Characterisation of Milling Fractions

**Figure 1:**
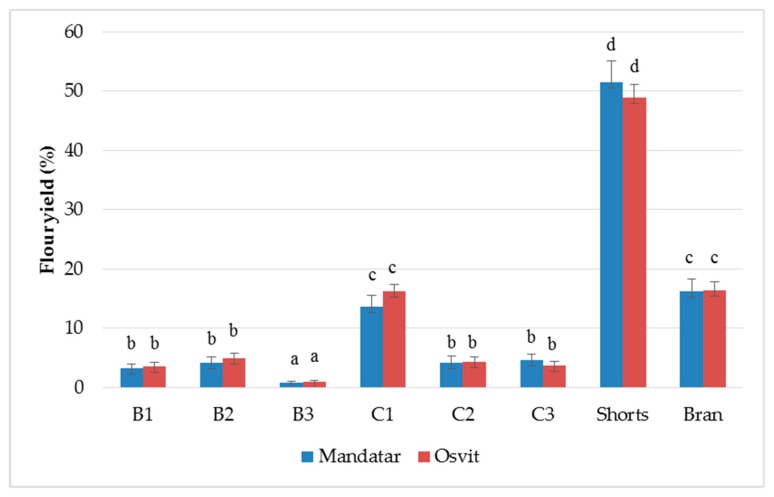
Relative yields (%) of break (B1–B3), reduction (C1–C2), shorts, and brans of hull-less barley. Different letters above columns indicate significant differences based on Fisher’s test (α = 0.05) for each cultivar.

The hull-less barley samples have been roller-milled using a Bühler MLU 202 laboratory mill, and three breaks (B1–B3), three reduction flour fractions (C1–C3), shorts, and bran were collected in total. Results showed that among C and B fractions, the yield varied from 0.79% (B3, Mandatar) to 16.27% (C1, Osvit) (Figure 1). The yield of the shorts was the largest (51.54% for Mandatar and 48.87% for Osvit), while the bran yield was 16.30% for Mandatar and 16.41% for Osvit. Andersson et al. [29] milled four hulls-less barley using the same laboratory mill and noted the highest yields of shorts (47–56%) and smaller amounts of bran (5–8%) also. Traditional roller milling fractionation of barley grain has not been applied and implemented as has been for wheat grain, and the fractions derived by this process of barley fractionation have not yet been standardised in terms of quality, composition, or terminology. Due to its heterogeneous composition, fractionation of hull-less barley kernel results in several products enriched through various constituents [25,40]. Izydorczyk et al. [27], in their study of milling hull-less barley with roller mill, noted that coarse material from the final reduction passage, designated as shorts, consisted mainly of fragments containing endosperm cell walls, with a smaller proportion of the outer grain tissues (39–49% yield), while in wheat milling, it comprised a fine bran. The “shorts” collected in the final step were designated as barley “fibre-rich fractions” (FRF). Light microscopy with differential staining revealed that some of the fragments contained considerable amounts of starch granules entrapped within endosperm cells. Additional refinement of the barley shorts fraction, which included pin milling and shorts duster passages, clearly reduced their particle size as well as removed a large proportion of the entrapped starch granules. Barley FRF can be further improved, but the process requires additional fragmentation of fibre particles by pin milling to dislodge adhering starch granules, and another shorts duster passage to separate the freed granules from the cell wall material. It resulted in enrichment of this product in β-glucans, arabinoxylans, proteins, and ash, a considerable decline of starch content, where the refinement had an influence on yield reduction of this fraction also. Barley fractions obtained through the additional purification steps were designated as “enriched fibre-rich fractions”, which has compositional complements and functional properties as well-known market oat bran [26,27,28].

**Figure 2:**
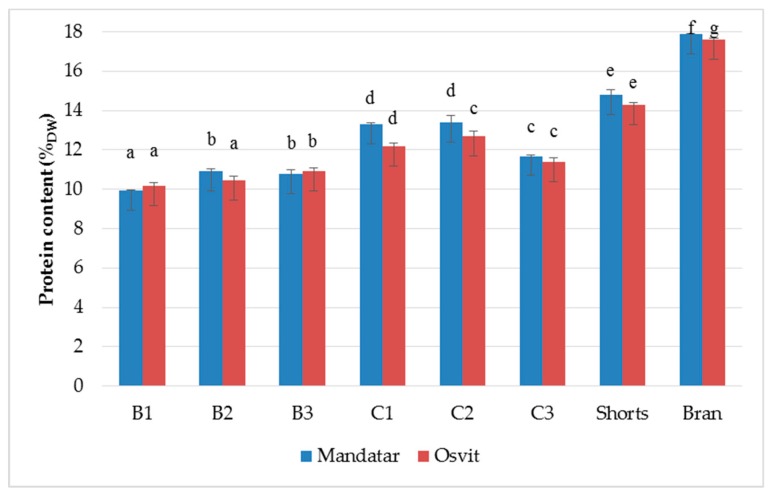
Protein content (%_dw_) of break (B1–B3), reduction (C1–C2), shorts, and brans of hull-less barley. Different letters above columns indicate significant differences based on Fisher’s test (α = 0.05) for each cultivar.

Milling products are often qualified according to their ash and grain protein content. They are important quality control parameters in the milling industry applicable to final flour products before putting them on the market. Based on literature findings, it is often assumed that barley bran, like wheat bran, consists of testa and pericarp, germ, aleurone, and the subaleurone layers. Barley bran is found to be brittle and difficult to separate from the endosperm by roller milling and, therefore, it increases the ash content and darkness of barley flour [40]. Results of the chemical composition of the different milling fractions showed that ash and protein contents were unevenly distributed along barley kernel (Table 1). Ash and protein content increased more from the endosperm fractions (B1–B3 and C1–C3) toward shorts and bran, while starch content decreased. Ash varied from 0.82% (B3, Mandatar) to 3.10% (Bran, Osvit). Protein content was the highest in the shorts (14.80%, Mandatar and 14.27%, Osvit) and bran (17.87, Mandatar and 17.6%, Osvit) (Figure 2), while starch content was the lowest in bran (48.69%, Mandatar) and the highest in the B3 fraction (82.31%, Mandatar and 81.90%, Osvit) (Table 1). The inverse distribution of chemical components obtained along the kernel parts of hull-less barley was confirmed by Andersson et al. [29].

Compared to starch and protein, the content of fat in barley is relatively low. Their contribution toward the nutritional value and storage stability of barley-based food or feed is important. Lahouar et al. [41] demonstrated that the main components of the unsaturated fatty acids in barley were oleic acid, linoleic acid, and α-linolenic acid. Distribution of fats among more endosperm fractions is clear (C1–C3 > B1–B3). The lowest content of fat was in B2 (1.50%, Mandatar) and B3 (1.56%, Osvit) fractions, while the highest content was obtained in the C2 fraction at both cultivars (2.96% and 3.26%, respectively). Shorts of Mandatar and Osvit had the content of fat on the same level as the C3 fraction (Table 1).

### 3.3. HPLC Albumins/Globulins and Hordeins

Siebenhandl-Ehn et al. [42] analysed 29 spring hull-less barley milled samples and reported that protein content varied from 8.7% to 13.3%. The major storage proteins in barley endosperm are hordeins (30–50%), whereas barley bran and germ are enriched in albumins and globulins (AG) as mainly cytoplasmic proteins with 3–4% and 10–20% in total protein, respectively [12].

On the basis of amino acid composition and molecular weights, hordeins (HORD) have been classified into three sub-fractions: high-molecular-weight prolamins (D), low-molecular-weight S-rich (B), and low-molecular-weight S-poor (C) [43]. Proline and glutamine are dominant amino acids in cereals, and in general, cereals have poor nutritional quality due to lower content of the most limiting amino acid lysine and tryptophan in the major storage proteins. C-hordeins have low cysteine content, which is the essential amino acid for developing disulphide bonds, but are rich in glutamine, proline, and phenylalanine. B-, D- and γ-hordeins have more Cys residues, which provide them with the possibility of developing inter and intra molecular disulphide bonds [12]. Shewry and Halford [44] noted that lysin content in barley protein fractions decreased in order AG > D-HORD > B-HORD > C-HORD (5.3, 1.1., 0.5, and 0.2 mol%, respectively)**.** Edney et al. [45] found that the concentrations of arginine, cysteine, isoleucine, lysine, methionine, and threonine were consistently higher in the hull-less barley samples than levels stated in the literature for barley. Sikdar et al. [46], using double-stranded RNAi silencing technology directed towards C-hordeins, obtained transgenic barley lines with up to 94.7% reduction of C-hordeins as a storage protein with the lowest nutritional quality, what may be a promising approach for improving the nutritional value of barley.

In contrast to the very clear distribution of total proteins in mill fractions at both cultivars (bran > shorts > C1–C3 > B1–B3), distribution of AG is not so clear. In both cultivars, AG content (0.78–1.41%) decreased in the order of shorts > B3 > bran, while AG distribution in other fractions was dependent on genotype. Distribution of HORD at both cultivars is at the same level, but, compared to AG, distribution of HORD between B and C flour fractions is reversed (Bran > B2–B3 > Shorts > B1 > C1–C3) (Table 2). In this study, proportions of the various HORD subfractions were quantified as a per cent of total HORD chromatogram peak area. Proportions of D-, C- and B- HORD sub-fractions in total HORD varied from 10.60% (Bran, Osvit) to 12.72% (B1, Mandatar), from 28.33% (B1, Osvit) to 30.24% (Bran, Mandatar), and from 58.36% (B3, Mandatar) to 60.07% (shorts, Osvit), respectively (Table 3). Such a quantitative relation of HORD sub-fractions in hull-less barley flour is consistent with our previous results (results not yet published). Considering the differences in the distribution of D-, C- and B-HORD sub-fractions, they are statistically irrelevant or non-existent for all fractions (Table 3). Šimić et al. [47] noted that barley grain protein content was highly significantly correlated with HORD content, but grain protein influence was not noticed on particular HORD subfractions proportions.

### 3.4. β-Glucan Content in Milling Fractions

**Figure 3:**
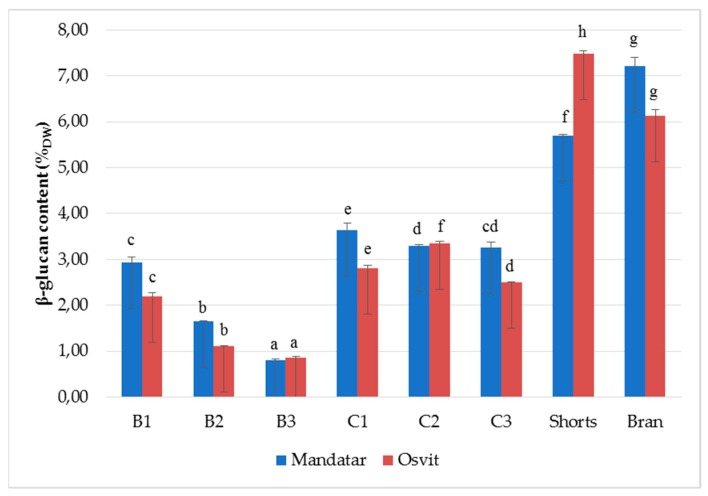
β-glucans (%) content in break (B1–B3), reduction (C1–C3), shorts and brans of hull-less barley. Different letters above columns of each cultivar indicate significant differences, Fisher’s test (α = 0.05).

Among cereals, barley and oat are known as the richest source of soluble and fermentable β-glucan [1], that possess protective and therapeutic effects against cardiovascular diseases, type-2 diabetes and certain cancers [48]. Recently, consumption of products containing grain constituents in the same proportions as in the native grains is recommended by nutritionists [49]. The β-glucan content in milling fractions is shown in Figure 3. The β-glucan content varied from 0.80 (B3, Mandatar) to 7.22% (Bran, Mandatar) and 7.49% (Shorts, Osvit). Differences in β-glucan content between fractions in both cultivars were strongly significant, and its value decreased in the order Bran, Shorts > C1–C3 > B1–B2 > B1. Moza and Gujral [6] reported that in the bran fraction, β-glucan content varied between 4.7% and 6.3%, while the refined flour fraction contained 3.4–4.4% of β-glucan. Wiege et al. [30] found that the highest β-glucan content was determined in bran fractions in two hull-less and two waxy barleys. Zheng at al. [50] noted that in the six hull-less barley cultivars, the shorts fraction contained the highest concentration of β-glucan (8.12–13.01%), followed by bran (6.15–7.58%) and flour (2.48–2.95%). According to the Martinez et al. [1] classification, shorts and bran, with 5–7% β-glucan content, could be classified as a moderate β-glucan flour, and with >7% as high β-glucan flour. Izydorczyk et al. [27] produced ’fibre-rich fraction’ (β-glucan levels from 8.83% to 18.19%) with additional refining of the hull-less barley shorts duster passages, which included pin milling, particle size reduction, and removal of a large proportion of the entrapped starch granules.

### 3.5. Total Phenolic Content and Antioxidant Activity of Milling Fractions

Phenolic compounds from barley are known to contribute to the antioxidant activity of cereal-based products. The total phenolic content (TPC) of different fractions ranged from 0.91 to 2.21 mg GAE/g_dw_ for Mandatar and 0.93 and 2.17 mg GAE/g_dw_ in Osvit, respectively. Phenolic content in the fractions was significantly different, but distribution was uneven and cultivar dependent. Their values decreased in the order Bran > Shorts > B2, C2 (Mandatar) > C3 (Osvit) > B1, C2 (Osvit) > B1 (Mandatar), B3 (Osvit) > C1, C3 (Mandatar), B3 (Osvit) > B2 (Osvit) > B3 (Mandatar) (Table 4). Moza and Gujral [6] reported the highest amount of total phenolic content in bran fraction (3.67–4.44 mg FAE/g_dw_), while more refined flours comprised lower amounts ranged between 1.30 and 1.61 mg FAE/g_dw._ Also, these authors reported that total phenolic content significantly varied within cultivars, milling conditions and milling fractions. The antioxidant activities of hull-less barley fractions evaluated by DPPH radical scavenging assay varied significantly in the range from 29.37% to 72.06% in Mandatar and 28.81% to 67.91% in Osvit and were also cultivar dependent and unevenly distributed (Table 4).

### 3.6. Phenolic Acids in Milling Fractions

In order to get a better insight into antioxidant activities of the hull-less barley milling fractions, the content of free and bound phenolic acids was determined using the HPLC method. In the free forms, predominant phenolic acids were p-hydroxybenzoic (3.43–17.44 ug/g_dw_), gallic (3.50–32.90 ug/g_dw_), and vanillic (3.20–24.70 ug/g_dw_) acids (Table 5). In recent studies, Arigò et al. [51] found that the most abundant free polyphenols compounds in barley were p-hydroxybenzoic acid, epicatechin, vanillic, syringic, and ferulic acids. Yang et al. [52] published that gallic and benzoic acid were dominant free phenolic acids. In this study, ferulic acid was not identified, while protocatechuic (3,4-dihydroxybenzoic) and syringic acids were found only in some fractions, and in average, below the quantification limit. Bran, except for vanillic acid in Osvit, had the highest content of free phenolic acids (15.35–32.90 ug/g_dw_). Distribution of free phenolic acids along the other fractions was uneven and variety dependent (Table 5).

**Figure 4:**
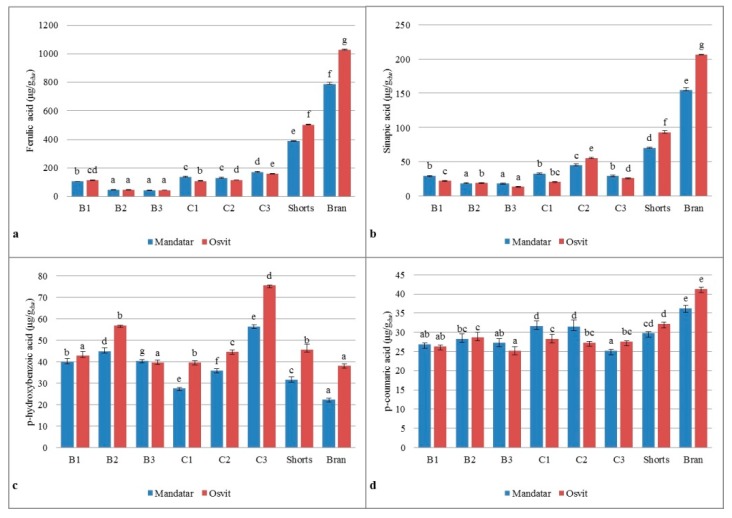
Bound phenolic acids: (**a**) ferulic, (**b**) sinapic, (**c**) p-hydroxybenzoic, (**d**) p-coumaric content in break (B1–B3), reduction (C1–C2), shorts, and brans of hull-less barley. Different letters above columns of each variety indicate significant differences; Fisher’s test (α = 0.05).

Considering bound phenolic acids, ten phenolic acids were identified (p-hydroxybenzoic acid, protocatechuic, gallic, vanillic, caffeic, syringic, p-coumaric, ferulic, sinapic, and o-coumaric), and only dominant ones were shown in Figure 4. The predominant ferulic and sinapic acid varied in the range from 45.16 to 1026.91 ug/g_dw_ and from 18.93 to 206.52 ug/g_dw_, respectively, with the highest content in bran and shorts. Arigò et al. [51] and Martínez et al. [1] noted that the major phenolic acids in hull-less and hulled barley are mainly ferulic (trans and cis-isomer), diferulic and trifeluric acid, p-hydroxybenzoic, p- and m-coumaric, vanillic, sinapic, 3,4-dihydroxybenzoic acid, and syringic acid. Distribution of ferulic acid as a major contributor to the antioxidant activity was unevenly balanced within decreased order from Bran > Shorts > C3–C1 > B1 > B2–B3. Many authors confirmed that ferulic acid distribution in cereals varied within the kernel where the highest content remains in the outer layers and lowest in the endosperm [53,54,55].

### 3.7. Principal Component Analysis of All Data

**Figure 5:**
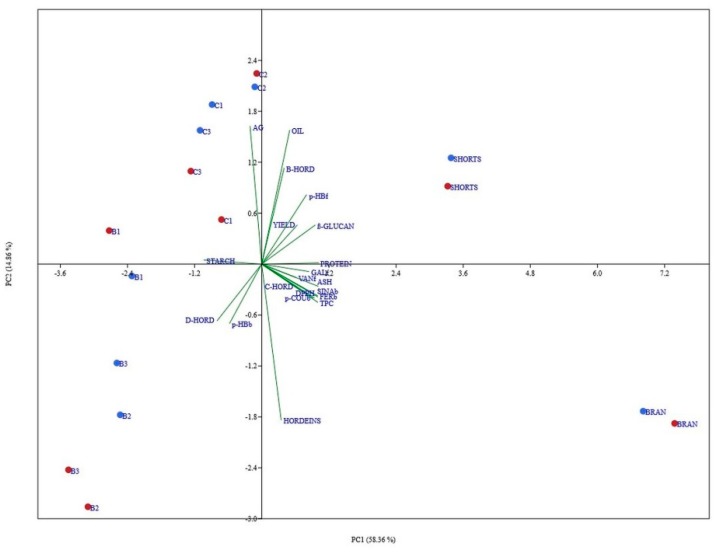
Projections of the variables onto the first two principal components.

The results obtained from PCA are a summary of all features, and both evaluated hull-less barley varieties and give us additional information about the aim of this study. Principal component analysis (PCA) was chosen to test whether the observed differences in milling fractions were genotype-specific and to what extent, and also to see the correlation of the above-mentioned fractions towards measured parameters. The first two PCs explained 73.22% variation present in the dataset (Figure 5). Shorts fraction for both varieties was present in the first quadrant, positive in both principal components, mostly correlated with flour yield, protein content, β-glucan content, etc. Also, trait-specific and varietal grouping was observed among other flour fractions, where Bran is grouped in the fourth quadrant, B fractions in third, and the C fractions in the second. PC analysis revealed a clear connection between different flour fractions and specific traits (green vectors), whereas this fact alone further affirms the logic behind univariate results mentioned before. Parameters of antioxidant activity (free and bound phenolic acids, TPC; DPPH) correlate positively with ash, proteins, and C-HORD and negatively with starch. The position of B glucan is expected given the chemical parameters, but it is interesting to note the positive correlation of this property with the antioxidant parameters and negative relation with D-HORD.

## 4. Conclusions

The response of the two hull-less barley varieties in terms of chemical and phytonutrients content and distribution was similar. Significant differences in phytonutrients distribution were found between outer layers and more endosperm fractions. Shorts fraction, which mainly originates from starchy endosperm cells with smaller outer layers, with the highest yield and elevated content of protein, starch, β-glucans, phenolic acids, and antioxidant activity has potential as a functional food ingredient. The results of this study could help in further research regarding milling performance of hull-less barley and factors governing milling quality, and to promote hull-less barley flours and enhance its limited use in commercially available quantities.

## Figures and Tables

**Table 1:** Chemical composition of milling fractions of two hull-less barley varieties.

Fraction	Ash (%)		Starch (%)		Oil (%)	
	Mandatar	Osvit	Mandatar	Osvit	Mandatar	Osvit
B1 *	1.06 ^b,^** ± 0.03	1.33 ^c,^** ± 0.01	77.75 ^d^ ± 0.86	78.23 ^e^ ± 0.27	1.80 ^b^ ± 0.06	1.41 ^a^ ± 0.07
B2	0.85 ^a^ ± 0.02	0.92 ^a^ ± 0.02	80.78 ^e^ ±0,67	79.38 ^e^ ± 1.08	1.50 ^a^ ±0.09	2.96 ^cd^ ± 0.12
B3	0.82 ^a^ ± 0.02	0.84 ^a^ ± 0.04	82.31 ^e^ ± 0.25	81.90 ^f^ ± 0.92	1.83 ^b^ ± 0.12	1.56 ^ab^ ± 0.14
C1	1.18 ^c^ ± 0.03	1.31 ^c^ ± 0.05	75.66 ^cd^ ± 1.06	74.84 ^cd^ ± 0.44	2.45 ^c^ ± 0.15	2.31 ^bc^ ± 0.10
C2	1.29 ^d^ ± 0.01	1.27 ^c^ ± 0.02	74.84 ^c^ ± 0.79	73.48 ^c^ ± 0.44	2.90 ^d^ ± 0.20	3.26 ^d^ ± 0.18
C3	1.05 ^b^ ± 0.03	1.13 ^b^ ± 0.04	75.19 ^cd^ ± 0.37	75.94 ^d^ ± 0.56	2.70 ^d^ ± 0.15	2.63 ^cd^ ± 0.19
Shorts	1.71 ^e^ ± 0.04	2.09 ^d^ ± 0.05	55.74 ^b^ ± 0.55	59.10 ^b^ ± 0.28	2.75 ^d^ ± 0.12	2.41 ^c^ ± 0.23
Bran	2.88 ^f^ ± 0.06	3.10 ^e^ ± 0.06	48.69 ^a^ ± 0.38	49.10 ^a^ ± 0.32	2.27 ^c^ ± 0.07	2.39 ^c^ ± 0.13

*B1–B3 (break fractions), C1–C3 (reduction fractions). ** Values are mean ± standard deviations. Different letters in columns indicate significant differences; Fisher’s test (α = 0.05).

**Table 2:** Content of albumine/globulins (AG) and total hordeins (HORD) in hull-less barley milling fractions.

Fraction	AG (g/100 g_dw_)		HORD (g/100 g_dw_)	
	Mandatar	Osvit	Mandatar	Osvit
B1 *	0.92 ^ab,^** ± 0.07	1.27 ^f^ ± 0.09	7.39 ^c^ ± 0.18	8.07 ^c^ ± 0.16
B2	0.95 ^ab^ ± 0.09	0.79 ^a^ ± 0.15	9.68 ^f^ ± 0.16	9.42 ^e^ ± 0.14
B3	0.96 ^b^ ± 0.23	0.87 ^b^ ± 0.16	9.09 ^e^ ± 0.09	9.29 ^e^ ± 0.12
C1	1.41 ^e^ ± 0.08	1.01 ^d^ ± 0.06	7.14 ^bc^ ± 0.08	7.23 ^b^ ± 0.18
C2	1.22 ^d^ ± 0.19	1.08 ^e^ ± 0.18	6.96 ^ab^ ± 0.03	6.78 ^a^ ± 0.05
C3	1.10 ^c^ ± 0.14	1.05 ^de^ ± 0.11	6.69 ^a^ ± 0.06	6.86 ^a^ ± 0.03
Shorts	1.14 ^c^ ± 0.26	0.95 ^c^ ± 0.19	8.19 ^d^ ± 0.20	8.39 ^d^ ± 0.16
Bran	0.90 ^a^ ± 0.20	0.78 ^a^ ± 0.12	9.76 ^f^ ± 0.19	10.49 ^f^ ± 0.22

* B1–B3 (break fractions), C1–C3 (reduction fractions). ** Values are mean ± standard deviations. Different letters in columns indicate significant differences, Fisher’s test (α = 0.05).

**Table 3:** Proportion * of D-, C- and B- HORD in hull-less barley milling fractions.

Fraction	D-HORD (%) *		C-HORD (%)		B-HORD (%)	
	Mandatar	Osvit	Mandatar	Osvit	Mandatar	Osvit
B1 **	12.72 ^c,^*** ± 0.02	12.26 ^c^ ± 0.04	28.43 ^a^ ± 0.14	28.33 ^a^ ± 0.10	58.86 ^a^ ± 0.12	59.42 ^abc^ ± 0.20
B2	12.59 ^bc^ ± 0.11	12.65 ^c^ ± 0.09	29.05 ^ab^ ± 0.25	29.06 ^ab^ ± 0.24	58.37 ^a^ ± 0.26	58.29 ^a^ ± 0.14
B3	12.28 ^b^ ± 0.12	12.31 ^c^ ± 0.19	29.36 ^ab^ ± 0.33	29.16 ^ab^ ± 0.19	58.36 ^a^ ± 0.15	58.53 ^ab^ ± 0.34
C1	12.48 ^bc^ ± 0.06	11.46 ^b^ ± 0.08	28.96 ^ab^ ± 0.15	29.57 ^b^ ± 0.41	58.57 ^a^ ± 0.26	58.97 ^abc^ ± 0.21
C2	11.48 ^a^ ± 0.07	11.37 ^b^ ± 0.03	29.31 ^ab^ ± 0.28	29.13 ^ab^ ± 0.22	59.20 ^a^ ± 0.32	59.50 ^abc^ ± 0.09
C3	11.34 ^a^ ± 0.01	11.44 ^b^ ± 0.04	29.60 ^ab^ ± 0.45	29.59 ^b^ ± 0.35	59.06 ^a^ ± 0.41	58.97 ^abc^ ± 0.19
Shorts	11.34 ^a^ ± 0.10	11.49 ^b^ ± 0.06	29.62 ^ab^ ± 0.19	28.73 ^ab^ ± 0.10	59.03 ^a^ ± 0.19	60.07 ^c^ ± 0.45
Bran	11.32 ^a^ ± 0.04	10.60 ^a^ ± 0.01	30.24 ^b^ ± 0.39	29.71 ^b^ ± 0.26	58.44 ^a^ ± 0.18	59.70 ^bc^ ± 0.49

* Proportion (%) of D-, C- i B- hordein fractions calculated as a per cent of total peak area; ** B1–B3 (break fractions), C1–C3 (reduction fractions). *** Values are mean ± standard deviations. Different letters in columns indicate significant differences, Fisher’s test (α = 0.05).

**Table 4:** Total phenolic content and antioxidant activity of hull-less barley milling fractions.

Fraction	Total Phenolic Content (mg GAE/g_dw_)		DPPH Activity (%)	
	Mandatar	Osvit	Mandatar	Osvit
B1 *	1.04 ^b,^** ± 0.01	1.05 ^bc^ ± 0.01	31.52 ^bc^ ± 0.06	31.27 ^b^ ± 0.22
B2	1.15 ^c^ ± 0.03	0.93 ^a^ ± 0.01	30.77 ^b^ ± 0.09	37.34 ^c^ ± 0.31
B3	0.91 ^a^ ± 0.01	1.02 ^b^ ± 0.02	34.70 ^d^ ± 0.18	36.19 ^c^ ± 0.38
C1	1.02 ^b^ ± 0.02	1.04 ^b^ ± 0.01	32.71 ^c^ ± 0.21	28.81 ^a^ ± 0.25
C2	1.18 ^d^ ± 0.05	1.05 ^bc^ ± 0.02	35.64 ^d^ ± 0.34	32.58 ^b^ ± 0.19
C3	1.02 ^b^ ± 0.01	1.09 ^c^ ± 0.03	29.37 ^a^ ± 0. 28	32.09 ^b^ ± 0.24
Shorts	1.59 ^e^ ± 0.04	1.57 ^d^ ± 0.04	70.43 ^e^ ± 0.75	65.38 ^d^ ± 0.69
Bran	2.21 ^f^ ± 0.03	2.17 ^e^ ± 0.05	72.06 ^f^ ± 0.62	67.91 ^e^ ± 0.55

* B1–B3 (break fractions), C1–C3 (reduction fractions). ** Values are mean ± standard deviations. Different letters in columns indicate significant differences, Fisher’s test (α = 0.05).

**Table 5:** Mean free phenolic acids (ug/g_dw)_ content in hull-less barley milling fractions.

Fraction	P-Hydroxybenzoic Acid		Gallic Acid		Vanilic Acid	
	Mandatar	Osvit	Mandatar	Osvit	Mandatar	Osvit
B1 *	10.24 ^b,^** ± 0.21	4.23 ^b^ ± 0.23	4.62 ^a^ ± 0.15	nd ***	7.38 ^c^ ± 0.15	3.2 ^a^ ± 0.09
B2	8.68 ^b^ ± 0.18	3.43 ^b^ ± 0.16	nd	3.5 ^a^ ± 0.12	3.57 ^a^ ± 0.02	nd
B3	8.78 ^b^ ± 0.12	nd	nd	6.1 ^c^ ± 0.09	5.29 ^b^ ± 0.18	nd
C1	13.79 ^c^ ± 0.18	4.39 ^b^ ± 0.17	5.72 ^b^ ± 0.16	4.0 ^ab^ ± 0.11	3.30 ^a^ ± 0.15	22.5 ^c^ ± 0.32
C2	13.83 ^c^ ± 0.24	12.55 ^d^ ± 0.41	4.50 ^a^ ± 0.19	27.3 ^d^ ± 0.25	3.39 ^a^ ± 0.20	nd
C3	13.45 ^c^ ± 0.09	7.89 ^c^ ± 0.15	5.35 ^ab^ ± 0.12	4.0 ^ab^ ± 0.17	7.69 ^c^ ± 0.08	3.5 ^a^ ± 0.14
Shorts	15.71 ^d^ ± 0.19	16.09 ^f^ ± 0.33	18.47 ^c^ ± 0.28	4.5 ^b^ ± 0.16	7.12 ^c^ ± 0.11	10.3 ^b^ ± 0.13
Bran	17.44 ^e^ ± 0.26	15.35 ^e^ ± 0.21	28.07 ^d^ ± 0.35	32.9 ^e^ ± 0.32	24.7 ^d^ ± 0.41	10.2 ^b^ ± 0.06

* B1–B3 (break fractions), C1–C3 (reduction fractions). ** Values are mean ± standard deviations. Different letters in columns indicate significant differences, Fisher’s test (α = 0.05). *** nd (not detected)
